# Clinical and inflammatory response to bloodstream infections in octogenarians

**DOI:** 10.1186/1471-2318-14-55

**Published:** 2014-04-23

**Authors:** Jessica Emily Green, Yohanes Ariathianto, Si Mun Wong, Craig Aboltins, Kwang Lim

**Affiliations:** 1 Austin Health, 145 Studley Rd, Heidelberg 3084, Australia; 2 Department of Medicine and Geriatrics, Northern Health, The Northern Hospital, 185 Cooper St, Epping 3076, Australia; 3 Aged Care Department, Eastern Health, Box Hill Hospital, Nelson Rd, Box Hill 3128, Australia; 4 Department of Infectious Diseases and Internal Medicine, Northern Health, University of Melbourne, The Northern Hospital, 185 Cooper St, Epping 3076, Australia; 5 Department of Medicine and Geriatrics, Northern Health, University of Melbourne, The Northern Hospital, 185 Cooper St, Epping 3076, Australia

**Keywords:** Bacteraemia, Sepsis, Inflammatory response, Octogenarians

## Abstract

**Background:**

Given the increasing incidence of bacteraemia causing significant morbidity and mortality in older patients, this study aimed to compare the clinical features, laboratory findings and mortality of patients over the age of 80 to younger adults.

**Methods:**

This study was a retrospective, observational study. Participants were taken to be all patients aged 18 and above with confirmed culture positive sepsis, admitted to a large metropolitan hospital in the year 2010. Measurements taken included patient demographics (accommodation, age, sex, comorbidities), laboratory investigations (white cell count, neutrophil count, C-reactive protein, microbiology results), clinical features (vital signs, presence of localising symptoms, complications, place of acquisition).

**Results:**

A total of 1367 patient episodes were screened and 155 met study inclusion criteria. There was no statistically significant difference between likelihood of fever or systolic blood pressure between younger and older populations (p-values of 0.81 and 0.64 respectively). Neutrophil count was higher in the older cohort (p = 0.05). Higher Charlson (*J Chronic Dis***40**(5)**:**373–383, 1987) comorbidity index, greater age and lower systolic blood pressure were found to be statistically significant predictors of mortality (p-values of 0.01, 0.02 and 0.03 respectively).

**Conclusion:**

The findings of this study indicate older patients are more likely to present without localising features. However, importantly, there is no significant difference in the likelihood of fever or inflammatory markers. This study also demonstrates the importance of the Charlson Index of Comorbidities (*J Chronic Dis***40**(5)**:**373–383, 1987) as a predictive factor for mortality, with age and hypotension being less important but statistically significant predictive factors of mortality.

## Background

As the population ages and life expectancy improves, infectious diseases have emerged as a serious problem causing significant morbidity, mortality and frequent hospital admissions in older patients. The incidence of bacteraemia increases significantly with age, with one study demonstrating greater than a threefold increase in the incidence of bacteraemia in the over 80 year old group compared to the 60–79 year old cohort [1].

Bacteraemia in older patients is also associated with a very poor prognosis. The mortality rate of 20-40% has not improved in 30 years despite the advent of new diagnostic modalities and antimicrobial therapy [2]. In addition, bactaeremic sepsis often results in functional decline and loss of independence.

There is much conflicting evidence regarding haematological and symptomatic responses in older patients who develop septicaemia [1,3]. It has been a largely held belief that older patients mount a poorer clinical and pathological immune response than their younger counterparts. It is thought that older patients with bactaeremic sepsis are more difficult to diagnose due to non-specific presentations such as fatigue, loss of appetite, falls, altered mental state, blunted febrile response [4,5] and absence of leucocytosis [6,7].

A number of studies do suggest that the ageing process alters the immune response, a phenomenon referred to as immunoscenescence [2]. Immunosenescence, or age-related defects in the human immune system affect the adaptive immune response as evidenced by major defects in cell-mediated immunity and by significant impairment of humoral immune responses with age. Yet despite this, there are limited valid studies comparing the clinical or haematological characteristics of older and younger adult populations [8].

It is thought that older patients are more likely to present in an ‘atypical’ or non-specific way with symptoms such as anorexia and weight loss, functional decline, falls and urinary incontinence [9,10]. Due to the perceived lack of clinical and laboratory indicators of sepsis, there is a risk of misdiagnosis, hence mistreatment in older patients who do not actually have sepsis. Thus it is important to better clarify the expected presentation of bacteraemia in older patients in order to more accurately diagnose and to more appropriately manage the patient’s condition.

A retrospective, observational study was conducted to determine if the presentation of older people with culture positive septicaemia is different form younger cohorts. The study compared clinical and pathophysiological factors of all adult patients admitted to a hospital for bacteraemia in the year 2010. The study also looked at predictors of mortality and the expected demographic of patients who develop blood culture positive bacteraemia.

## Methods

### Study population

The study inclusion criteria were adults (greater than 18 years of age) who were admitted to The Northern Hospital, a metropolitan teaching hospital in suburban Melbourne over the period of January 1st 2010 to December 31st 2010 inclusive. A single patient episode was defined as the first positive blood culture result for a single patient in a single admission. Subsequent positive blood culture results for an individual patient during the same admission were disregarded. Patients were excluded from the study if their blood culture specimen contained likely contaminants, or if they were undergoing anti-tumour chemotherapy. Contaminants were defined as organisms grown such as Staph coagulase negative, where there were no clinical features of an infection and the growth of the organism was likely to be a result of a contaminated collection process. Blood culture contaminants were further reviewed on a case by case basis by file review by an infectious diseases physician. Anti-tumour chemotherapy patients were excluded as they are immunosuppressed and often do not grow organisms despite high fevers.

Patients were also excluded from the study if they were transferred to a different hospital or facility during the acute stage of their illness as data was incomplete for these patients. Patients were analysed as two groups: the younger cohort (age18-80) and older cohort (age >80).

### Data collection

Ethics approval was obtained from the Northern Health Human and Research Ethics Committee. Positive blood culture results were obtained from the hospital’s pathology department database. All positive blood culture results from 2009 to 2011 were screened, should any patient episode from the year before and after have overlapped with the year 2010. Thus 1367 patient episodes were screened. Further patient data was obtained retrospectively from the hospital medical records. Several variables were recorded including source of infection, clinical, personal and pathological factors.

Place of acquisition was divided between community acquired, hospital acquired and care facility acquired. Hospital acquired infection was defined as culture positive bacteraemia arising more than 48 hours into a hospital admission. Care facility acquired bacteraemia was defined as culture positive bacteraemia arising in a resident of a care facility within 48 hours of hospital admission. Community acquired bacteraemia was defined as culture positive bacteraemia arising within 48 hours of hospital admission in a patient not living in a care facility [5]. Fever was defined as a temperature of greater than 37.2° Celsius (98.96° Fahrenheit) [11]. Localising symptoms were decided by taking the source of sepsis as stated in the discharge summary and retrospectively reviewing the notes for corresponding localising symptoms (i.e. dysuria in urosepsis).

### Data analysis

Researchers who collected the data did not analyse it. Data was analysed in two subsets: younger cohort (age18-80) and older cohort (age >80). Data was analysed using intention to treat analysis. T-tests were used to compare means, chi squared tests to compare proportions and multivariate analysis was performed using logistic regression analysis. Results were interpreted using SPSS statistical software. The reference model used was IBM Corp. Released 2011. IBM SPSS Statistics for Windows, Version 20.0. Armonk, NY: IBM Corp

## Results

### Selection of study population

**Figure 1:**
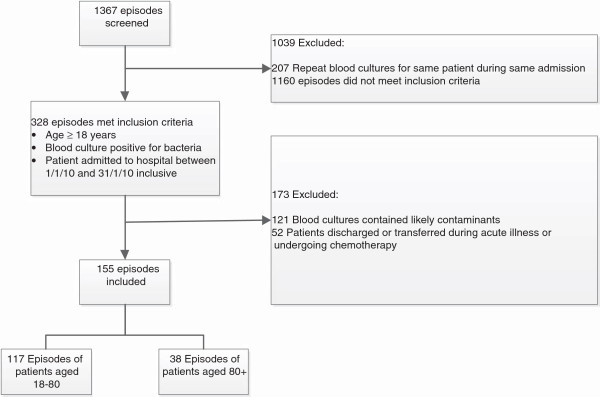
Selection of patient study population.

A total of 1,367 patients were screened and of these, 328 episodes met the study inclusion criteria. The list of patients screened was taken from the hospital database and included patient data from years 2009–2011 inclusive. Most patients from this cohort were excluded because they fell outside of the study period (January 1^st^ to December 31^st^ 2010). The reason it was necessary to screen such a large number of patients from the years surrounding the index year as not to miss patient episodes which overlapped with the index year but commenced or began in the years preceding. The remaining episodes screened did not meet the inclusion criteria because they were under 18 years of age, their blood cultures were not positive for bacteria or they were repeat blood cultures for the same patient during the same admission. A further 173 were excluded bringing the total number of eligible patient episodes to 155. Of those included in the study, 117 episodes included patients aged between 18 and 80 years of age and 38 episodes included those aged 80 years or above (see Figure 1).

### Baseline demographics

With respect to demographics, the mean age across both patient groups was 63 years of age. Patients aged 80 years and above represented around one quarter of the total study population (24.5%) with a mean age of 87 years (standard deviation of 3.9). Patients aged between 18 and 80 years of age represented the remaining three quarters (75.5%) of the sample size with a mean age of 55 years (standard deviation 16.9) for the younger cohort. The proportion of males to females was similar in both age groups. Men formed the majority in both groups with an overall proportion of 59.4% males in the study. A large majority of patients came from home, 83.9% overall. However, more patients came from high level care nursing homes than low level care nursing homes 11% and 5.2% respectively (see Table 1).

**Table 1:** Patient demographics

**Characteristic**		**Overall: n (%)**	**Patient group: Age ≥80, n (%)**	**Patient group: Age <80, n (%)**
Mean age		63 (100)	87.2 (24.5)	58.8 (75.5)
Female		63 (40.6)	14 (36.8)	49 (41.9)
Male		92 (59.4)	24 (63.2)	68 (58.1)
Regular accommodation	LLC	8 (5.2)	6 (15.8)	8 (1.7)
	HLC	17 (11.0)	10 (26.3)	17 (6)
	Home	130(83.9)	22 (57.9)	130 (83.8)
Place of acquisition	Community	117 (75.5)	19 (50)	98 (83.8)
	Hospital	14 (9)	4 (10.5)	10 (8.5)
	Aged care facility	24 (15.5)	15 (39.5)	9 (7.7)
Total		155 (100)	38 (24.5)	117 (75.5)

n – Number of patients.

LLC – Low Level Care Facility.

HLC – High Level Care Facility.

Place of acquisition was most commonly community acquired, 75.5% overall. However, the likely source of infection varied significantly between the two age groups with 83.8% of younger patients presenting with likely community acquired sepsis compared to only 50% in the older group. In the older patient group, aged care facility acquired infection represented 39.5% of episodes. Proportion of hospital acquired infection was similar in younger and older populations with 10.5% and 8.5% respectively.

### Mortality

The incidence of mortality was 11.6% overall. There was a statistically significant difference between the two cohorts (p = 0.04) of 21.1% in the older group compared to 8.5% in the younger cohort.

Corrected for comorbidities however, the difference between the two age groups was minimal with an odds ratio of only 1.05 for advanced age. Increased Charlson [12] Index of Comorbidities was also found to be the most important statistically significant predictors of mortality. Lower systolic blood pressure was also found to be a statistically significant predictor of mortality (p = 0.06). Source of infection, greater respiratory rate and lower oxygen saturation were not significant predictors of mortality (see Table 2).

**Table 2:** Logistic regression of predictors of mortality according to patient variables

	**Odds ratio**	**95% Confidence Interval**	**P-value**
Charlson Index of Comorbidities	1.36	1.05-1.75	0.02
Age	1.05	1.01-1.10	0.02
Systolic Blood pressure	0.97	0.95-1.00	0.03
Oxygen Saturation	0.99	0.96-1.01	0.27
Respiratory rate	1.06	0.98- 1.14	0.15

### Clinical and laboratory findings

There no significant difference in the proportion of gram positive or gram negative bacteraemia between the two age groups. The younger population group was found to have a greater likelihood of developing localising symptoms. 68.4% of the younger population developed localising symptoms compared to 55.3% of older patients (p = 0.00).

Importantly, there was no statistically significant difference between the two groups in terms of likelihood of developing fever (p = 0.96). Nor was there a difference in systolic blood pressure or respiratory rate. However, there was a difference in heart rate with the younger group having an average heart rate of 12 beats per minute greater than the older group (p = 0.01). Oxygen saturation was found to be an average of 5% lower in the younger population compared to the older group (p = 0.03).

There was no significant difference in inflammatory markers between the two groups. However, neutrophil count approached significance with an average difference of 3x10^9^/L greater in the older group (p = 0.06) (see Table 3).

**Table 3:** Clinical presentation and laboratory measurements of patients presenting with culture positive septicaemia

		**Patient group: Age ≥80**	**Patient group: Age <80**	**p-value**
Presence of localising symptom, n (%)		21 (55.3)	80 (68.4)	0.00
Presence of fever, n (%)		30 (78.9)	93 (79.5)	0.96
Vital signs, mean value (s.d.)	Temperature	38.0 (1.1)	38.1 (1.6)	0.96
	Systolic blood pressure	115 (29)	113 (26)	0.70
	Heart rate	96 (18)	108 (26)	0.01
	Respiratory rate	23 (7)	23 (7)	0.57
	Oxygen saturation	93 (5)	88 (23)	0.03
Inflammatory markers, mean value (s.d.)	White cell count	15.7 (9.5)	13.1 (6.5)	0.13
	Neutrophil count	13.7 (8.5)	10.9 (6.0)	0.06
	C-reactive protein	198 (117)	157 (160)	0.09
Total		38 (24)	117 (76)	

n – number of patients.

s.d. – standard deviation.

### Organisms cultured

There were several statistically significant differences between the two groups on univariate analysis with logistic regression (Pearsons Chi Squared = 0.01). The following differences are statistically significant. Interestingly, although commonly cultured in the younger group (17.1%), only a single case of Methicillin Sensitive Staphylococcus Aureus was cultured in the older group. Escherichia Coli positive bacteraemia represented 26.5% of cases in the younger group, but almost double that in the older group with 42.1%. Strep Pneumonia represented only 3.4% of cases in the younger group but 13.2% of cases in the older group (see Table 4).

**Table 4:** Organisms cultured

	**Patient group: Age ≥80, n (%)**	**Patient group: Age <80, n (%)**	**Total, n (%)**	**p-value**
**Gram Positive Organisms**	14 (36.8)	56 (47.9)	70 (45.2)	0.24
MSSA	1 (2.6)	20 (17.1)	21 (13.5)	0.01
MRSA	1 (2.6)	1 (0.9)	2 (1.3)	0.01
Streptococcus Pneumonia	5 (13.2)	4 (3.4)	9 (5.8)	0.01
Other Streptococcus	6 (15.8)	19 (16.2)	25 (16.1)	0.01
Enterococcus	5 (13.2)	6 (5.1)	11 (7.1)	0.01
**Gram Negative Organisms**	24 (63.2)	61 (52.1)	85 (54.8)	0.24
Bacteroides	0 (0)	5 (4.3)	5 (3.2)	0.01
Klebsiella	0 (0)	4 (3.4)	4 (2.6)	0.01
Proteus species	0 (0)	3 (2.6)	3 (1.9)	0.01
Pseudomonas species	3 (7.9)	4 (3.4)	7 (4.5)	0.01
Escherichia Coli	16 (42.1)	31 (26.5)	47 (30.3)	0.01
**Other**	0 (0)	16 (13.7)	16 (10.3)	0.01
Total	38 (100)	117 (100)	155 (100)	

n – number of patients.

MSSA - Methicillin Sensitive Staphylococcus Aureus.

MRSA – Methicillin Resistant Staphylococcus Aureus.

E. Coli was the most commonly cultured organism overall representing 30.3% of all episodes. Strep Pneumonia, Other Strep and Enterococcus were similarly represented in the older group with 13.2%, 15.8% and 13.2% respectively. In the younger group Other Strep species represented 16.2% of positive blood cultures with Strep Pneumonia and Entorococcus being much less common.

## Discussion

Baseline body temperature is thought to decrease with ageing and increased frailty, even in the absence of infection [13-16]. This is likely attributable to multiple causes including biological changes with ageing, coexisting chronic disease and concurrent medications. In one study, a “blunted febrile response” was been described in nursing home residents wherein an increase in body temperature of 1.3°C above baseline warrants an assessment for the presence of infection [16]. This study examined nursing home residents only and did not include a younger control group. However, perhaps because of this study and others like it [13-15], it is thought that older patients are less likely than younger patients to mount a febrile response to infection or bacteraemia.

Our study however found no significant difference in the incidence of fever between the younger and older cohort. Importantly around 80% of older patients with bacteraemia presented with fever and that baseline temperature on average is about 38°C the older group and 38.1°C in the younger group (p = 0.96).

This study also showed a statistically significant decreased likelihood of tachycardia in older patients. This is likely due to the normal physiological changes of ageing. Both the intrinsic and maximum heart rate decreases with age leading to reduced cardiac output, inotropic and chronotropic response to beta adrenergic stimulation and reduced total T3 [17,18]. In contrast to a study by Iberti et al., this study did not find a statistically significant increase in the incidence of tachypnoea in the older cohort.

As with previous literature, this study demonstrated the absence of localising symptoms in older bactaeremic patients [4,5].

This study demonstrated that older patients do not mount a poorer immune response than younger patients. In fact, on average these patients had higher C-reactive protein, white cell count and neutrophil count. The difference in neutrophil count was on average three ×10^9^/L higher in older patients, a result very close to statistical significance (p = 0.06).

This study also demonstrated a similar total number of gram positive and gram negative organisms, gram negative organisms slightly more predominant (54.8% overall). There was a non-significant trend towards gram negative organisms in the older cohort and gram positive organisms in the younger group, which is consistent with previous reports [19,20]. E. Coli was the most commonly cultured organism overall representing 30.3% of all episodes. It was by far the most commonly cultured organism in the older age group.

Older patients are thought to be more susceptible to severe sepsis and its associated complications due to poor physiological reserve and the high prevalence of malnourishment. Thus it is thought that older sepsis survivors are likely to be left with significant functional decline and to require higher level of care including permanent residential care [3,21].

In this study however, advanced age was found to be a trivial predictor of mortality. When adjusted for comorbidities the odds ratio was only 1.05 (P = 0.02) for greater age. Instead this study demonstrated the importance of medical comorbidities as measured by Charlson [12] index as a prognostic factor in bacteraemia with an odds ratio of 1.36 (P = 0.02). It was also found that hypotension, but not hypoxaemia, at presentation is associated with increased mortality. Presumably, this is because hypotension signifies a systemic inflammatory response and septic shock with multi-organ involvement.

Other factors which were not examined in this study but have been found to have prognostic value include underlying cognitive status, functional ability, chronic urinary incontinence, elevated lactate dehydrogenase and hypoalbuminaemia [22]. In addition acute confusion and hypotension have been shown to carry a poorer prognosis. Delay in diagnosis and management, especially in commencing antibiotics have also been found to correlate with poor outcomes [23].

### Study limitations

The fact that only cases of culture positive bacteraemia were included in the study may be regarded as a limitation. However, considering that the clinical and laboratory variables used to diagnose culture negative sepsis were the very variables being examined by the study, clearly these cases could not be included.

Another potential limitation is the retrospective nature of the study. This meant researchers needed to rely upon clinical notes. However, to minimise the subjective and unclear information derived from the notes, results were interpreted and analysed as numbers and categorical variables only.

The sample size was relatively small in that only data from a single year was analysed. However significant results were obtained and thus further data analysis was not considered necessary.

## Conclusions

Bacteraemia is a common clinical scenario [1] thus an accurate, evidence based approach to diagnosis and management is of great importance. The findings of this study indicate that despite having decreased likelihood of localised symptoms, older patients have a similar inflammatory and laboratory presentation to younger patients. This study also demonstrates the importance of the Charlson Index of Comorbidities [12] as predictive factor for mortality with age and hypotension being less important but statistically significant predictive factors of mortality. Further research into this area, such as replication studies with greater patient numbers would be of great value in further clarifying and improving the understanding, diagnosis and management of bacteraemia in older patients.

## Competing interests

The manuscript, or parts of it, have not been and will not be submitted elsewhere for publication. The authors declare there is no conflict of interest. The study was funded by Northern Health.

## Authors' contributions

JG: Primary author, data entry and literature review. YA: Data entry, contribution to manuscript and literature review. SW: Primary drafting, primary data entry and literature review. CA: Interpretation of data and editing of manuscript. KL: Initiation and development of study design, editing of manuscript, interpretation and analysis of data. All authors read and approved the final manuscript.

## Authors' information

Dr Jessica Emily Green is an intern at The Northern Hospital.

Dr Yohanes Ariathianto is a geriatrician at The Northern Hospital.

Dr Si Mun Wong is a geriatrician at Box Hill Hospital.

Dr Craig Aboltins is an infectious diseases physician at Then Northern Hospital.

A/Prof. Kwang Lim is the Chief Medical Officer at The Northern Hospital and a geriatrician.

## Pre-publication history

The pre-publication history for this paper can be accessed here:

http://www.biomedcentral.com/1471-2318/14/55/prepub
